# The contribution of teacher, parental and peer support in self-reported school and general well-being among ethnic-cultural minority and majority youth

**DOI:** 10.3389/fpsyg.2022.1051143

**Published:** 2022-12-16

**Authors:** Barbara Valcke, Kim Dierckx, Laura Desouter, Stefan Van Dongen, Guido Van Hal, Alain Van Hiel

**Affiliations:** ^1^Department of Developmental, Personality and Social Psychology, Faculty of Psychology and Educational Sciences, Ghent University, Ghent, Belgium; ^2^Evolutionary Ecology Group, Department of Biology, Antwerp University, Wilrijk, Belgium; ^3^Social Epidemiology and Health Policy, Faculty of Medicine and Health Sciences, University of Antwerp, Wilrijk, Belgium

**Keywords:** teacher support, parental support, peer support, school well-being, general well-being, ethnic diversity

## Abstract

Social support has been shown to be a crucial element in the well-being of children and adolescents. The present research article investigated how various sources of social support (i.e., parental support, teacher support and peer support) are related to school well-being and general well-being,. A survey was administered to *N* = 12,215 primary school pupils, pertaining to three ethnic-cultural groups, i.e., the national majority group, the Eastern European minority group and the Middle Eastern minority group. The results showed that perceived teacher support was most strongly and positively related to school well-being, although peer support was also an important determinant of school well-being. All three sources of perceived support were positively related to general well-being. Furthermore, and contrary to previous research, no significant differences were found between both minority groups and the national majority in terms of perceived teacher support. Conversely, both minority groups reported lower perceived parental and peer support. It was further shown that minority status moderated the relationship between the various sources of support and school well-being, although it should be articulated that these effects sizes were fairly small. School diversity, finally, did not yield any relevant effects. Similarities and differences with the existing literature on school well-being are delineated, and potential explanations for these divergences are discussed.

## 1. Introduction

Educational research has revealed that cultural minority members often do worse than White students in terms of their academic achievements (e.g., Griffith, 2002; Kurtz-Costes et al., 2014; Musu-Gillette et al., 2016). This finding has been reported in the North American context, as well as on the international level. Test scores on the PISA test (i.e., Programme for International Student Assessment) for instance, have constantly revealed that cultural minority students obtain lower scores than Whites on each of the three included tests, that is, on mathematics (e.g., Schleicher, 2003), reading (e.g., Baumert and Schümer, 2001; Hvistendahl and Roe, 2004) and science (e.g., Schleicher, 2006). Furthermore, school performance of children in ethnically diverse classes has been reported to be lower than in less diverse classes (e.g., Mok et al., 2016). Often mentioned reasons for these regretful findings reside in, among many others, minority students’ lower social-economic status, lower language proficiency, and lower parental involvement, as well as in the fact that because of school segregation minority children flock together (Kurtz-Costes et al., 2014; Marks et al., 2018). In other words, in today’s multicultural society, these studies have revealed that it is interesting to look at both the individual students’ ethnic background characteristic on the one hand, and ethnic diversity as a school climate variable on the other hand, as explanatory variables of minority’s school performance.

While the study of school performance is undoubtedly interesting in its own right, the argument has also been made that it is important that students feel comfortable at school (Kessler et al., 1995; Rosenfeld et al., 2000). It should thus be acknowledged that school well-being, and the processes underlying it, are relevant outcomes (e.g., Furlong et al., 2003). In the current study, we investigate school well-being of cultural minority and majority pupils, and we will look at this issue on the individual level in terms of students’ perceived social support, as well as on the school level in terms of diversity. Specifically, in the current study we investigate if children’s school well-being is influenced by the perceived social support of parents, teachers and their peers, using a multi-level approach in which cultural group membership is included as an individual-level variable, and school diversity as the context-level variable. This research question is addressed in a West-European context among pupils aged 9–12 who complete primary school.

### 1.1. Subjective well-being and social support

In psychological research, “feeling good” is often measured as subjective well-being (e.g., Diener et al., 1999). Subjective well-being is defined as a construct consisting of three often substantially correlated aspects, namely affect, domain-specific satisfaction (e.g., work, relationships, …), and general life satisfaction (Diener et al., 1999). Diener et al. (1985) describe subjective life satisfaction as what one considers to be “a good life.” This form of subjective happiness can be described as “a global assessment of a person’s quality of life according to his own chosen criteria (Shin and Johnson, 1978; p. 478), or as “the harmonious satisfaction of one’s desires and goals” (Chekola, 1975). Subjective well-being can be associated with positive outcomes on a variety of aspects of life, such as higher odds of being married or employed, higher income (Marks and Fleming, 1999), and a reduced risk of cardiovascular disease (Boehm and Kubzansky, 2012).

Although there are many antecedents of well-being, social support is considered to be one of the more important bases. Indeed, social support has been shown to correlate strongly with psychological well-being (e.g., Cohen and Wills, 1985; Chou, 1999; Gallagher et al., 2008; Kong et al., 2013; Lee et al., 2018). Social support has been defined as communication from others that is being interpreted in terms of being cared for and loved, being esteemed and valued, and being part of a network of communication and mutual obligation. In other words, social support can be seen as a conglomerate of emotional support, recognition, and belongingness (see Cobb, 1976).

A distinction in support types has been provided by Helgeson (1993), namely emotional, instrumental and informational, and the various forms of aid and assistance comprised by the term social support can be supplied by different sources, e.g., family members, friends, neighbors, and others (Barrera et al., 1981). Furthermore, a distinction can be made between perceived support, i.e., the extent to which people believe support is available, and received support, i.e., the amount of support that has actually occurred (Helgeson, 1993). Cohen and Wills (1985) suggest that social support influences well-being through the provision of positive emotions and predictability in life, as well as through encouraging a positive self-concept.

The positive relationship between social support and psychological well-being has been reported in children as well (e.g., Rigby, 2000). Child well-being has been described as the outcome of the interaction between risks and resources available to children, thus constantly changing with both their evolving capacities as well as with the variations in their personal situation (Bradshaw et al., 2007). Moreover, as children have a social network that is substantially different from that of adults, both in terms of size (Wrzus et al., 2013) as well as in terms of the nature of relationships, i.e., more vertical than horizontal relationships with adults (Russell et al., 1998), social support may impact child well-being differently than adult well-being (e.g., Hartup, 1989).

In a meta-analysis of the relationship between social support and general well-being in children, Chu et al. (2010) showed that perceived support is associated more strongly with well-being than other measures of social support such as, for instance, enacted support and size of social network. Furthermore, these authors also reported differential effects of social support for different sources, and they particularly showed that social support of parents and of teachers and school personnel is especially important for well-being. Social support of friends was reported to have a somewhat limited effect. It stands to reason that the huge importance of social support for general well-being has been revealed for well-being in specific contexts as well, such as the school context. Perceived teacher support has been found to be important for school well-being of adolescents. Specifically, in a study on adolescents’ emotional, behavioral and cognitive school engagement, it was revealed that although family support is directly linked to school engagement, the greatest impact comes from perceived teacher support (Ramos-Diaz et al., 2016). More specifically, scores on measures of school related affect, like school satisfaction (measured with items such as “I enjoy going to this school” and “I like the classes that I am taking”) and school engagement (measured with items such as “I find school fun and exciting”) coincide with high perceived teacher support, and to a lesser extent, both high perceived parental and peer support (Rosenfeld et al., 2000). Gutiérrez et al. (2017) showed a direct effect of perceived teacher support on satisfaction with school, as well as an indirect effect through school engagement. A longitudinal study demonstrated that children with high teacher-child closeness and low teacher-child conflict reported higher school liking 2 years later (Wang et al., 2016). In the current study, we look at the contribution of perceived social support from teachers, as well as from parents and peers, on both general well-being and school well-being.

Based on the literature mentioned above, we formulate the following hypothesis:

*Hypothesis 1a*: Perceived support of parents, teachers and peers will all be positively related to well-being, both school and general wellbeing.

*Hypothesis 1b*: The relationship between perceived support and school well-being will be strongest for teacher support (compared to parental support and peer support).

*Hypothesis 1c*: The relationship between perceived support and general well-being will be strongest for parental support (compared to teacher support and peer support).

### 1.2. Social support in the context of minorities

Perceived teacher support has been shown to be an important factor in children’s academic development as well as in their school well-being (e.g., Roeser et al., 2000; Natvig et al., 2003; Danielsen et al., 2009; Roorda et al., 2011; Tian et al., 2015; Ruzek et al., 2016). In the present context, our aim is to answer two questions about social support. First, are there differences between majority and minority pupils in their perception of social support, in well-being, and does being part of the majority or minority group moderate the relationship between perceived social support and well-being? Second, are school diversity and class diversity relevant context variables in perceived social support and in well-being, and do these context variables moderate the relationship between perceived social support and well-being?

#### 1.2.1. The role of social support at school for minority group members

Research in the U.S. has indicated that minority students perceive teacher support somewhat differently than students pertaining to majority groups. Specifically, African American children have less positive relationships with their teachers (Hamre and Pianta, 2001; Hughes and Kwok, 2007), which may reflect lower levels of perceived teacher social support. It is nonetheless of particular importance for students of minority groups to have positive teacher relationships, and this has been shown by different studies. For African American students who were rated behaviorally at-risk by their teachers, Decker et al. (2007) reported that improvements in the student-teacher relationship correlate with more favorable social, behavioral and academic outcomes. Along similar lines, Murdock (1999) reported that for African American students from low-income homes who were at risk of dropping out of school, motivational context such as support of teachers was positively related to beneficial behavioral outcomes such as engagement in school tasks and a decrease in disciplinary problems. In a similar study using a sample of at-risk Latino students, although perceived parental support was also important, perceived teacher support had the largest effect on “liking school” (Brewster and Bowen, 2004). Furthermore, not only cultural minority students with problematic academic behavior can benefit from a positive relation with their teacher. The combination of school expressive support (i.e., students feel there are adults at school they can talk to about problems and who will take care of them when needed) and classroom instrumental support (i.e., students feel their teacher helps them in learning different subjects and provides them with feedback) has been shown to reduce the gap in academic achievement between minority and majority students (Griffith, 2002). Thus, perceived teacher support has been shown to be important for various outcomes in minority student groups.

In European samples, similar effects of teacher support on school-related outcomes in minority groups have been shown. A recent study by Göbel and Preusche (2019) using a German sample of minority students further demonstrated that perceived teacher support, but not perceived parental support, relates to emotional school engagement. Additionally, a Belgian study has shown that perceived support from teachers reduces the attainment gap between Turkish Belgian minority students and Belgian non-minority students (Baysu et al., 2016).

Seemingly contradictive to the findings of lower academic achievement and lower perceived teacher support in students of minority groups, previous studies have shown that cultural minority students are more satisfied with school (Verkuyten and Thijs, 2002). According to Verkuyten and Thijs (2002), this might be caused by a more positive attitude towards education in minority groups (Mickelson, 1990), and minority pupils might view the educational context as more liberal compared to more strict social control at home (Dagevos and Veenman, 1992). On this particular matter, we constructed one hypothesis and three research questions. For these research questions, we do not have *a priori* predictions about the effects, but are nonetheless interested in including these in our analyses in an exploratory fashion.

*Hypothesis 2*: Children of cultural minority groups will report lower perceived teacher support (compared to majority group pupils).

- Research question 1: What are the possible effects of cultural minority status on perceived peer and parental support?
- Research question 2: Do children of cultural minority groups report higher school well-being (compared to majority group pupils)?
- Research question 3: Does cultural minority status affect the relationship between perceived support (teacher, parental and peer) and school well-being?

#### 1.2.2. Diversity as a relevant context variable

To the best of our knowledge, only two multi-level analysis studies (Bottiani et al., 2016; Parris et al., 2018) have investigated the effect of student-level ethnicity and school-level diversity on perceived school support. The study by Bottiani et al. (2016) conceptualized perceived school support as perceived caring (i.e., students believe teachers care about them), high expectations (i.e., students believe their teachers have high academic expectations of them), and equity (i.e., students’ perceived fairness in how they are treated). We are specifically interested in the dimension of perceived caring, as this is closest to our measurement of perceived teacher support. With respect to this dimension, the authors found that perceived caring is lower in Black students, but not affected by school diversity. Furthermore, no cross-level effects were found for the caring dimension.

Another multi-level study that is relevant for the present purposes was conducted by Parris et al. (2018) who investigated the effect of ethnicity and diversity on school climate. School climate in this study is a composite variable measured by different subscales, including peer social support and adult social support (Parris et al., 2018). The results showed that perceptions of school climate were more positive for White students as compared to Asian, African American, and Hispanic students, and more negative when school diversity was at a higher level. Moreover, these authors also obtained a cross-level moderation effect of school diversity on the relationship between students’ ethnicity and perception of school climate. Higher diversity curbed school climate perceptions for all ethnic groups included in the study, but this trend was weaker for African American students. For the specific aspects of school climate, the moderation effect varied across specific cultural groups. For example, in terms of peer support, African American and Hispanic students reported a smaller decrease in more diverse schools, whereas White students reported a larger decrease, and Asians were largely unaffected by school diversity.

Furthermore, only few studies on the effects of school diversity have probed into emotional instead of academic outcomes, and these studies often focused on specific cultural groups. A body of research has shown the effects of school diversity for African-American and Latino children. These studies demonstrate, for example, that for youth of color greater school diversity is associated with lower social vulnerability, less peer victimization and loneliness, and a greater sense of safety (Juvonen et al., 2006; Graham et al., 2014). Moreover, studies have shown that perceived institutional discrimination reported by minority students is higher when the school setting is less diverse, and that this is associated with lower life satisfaction (Seaton and Yip, 2009). Conversely, Benner and Graham (2013) reported that in students of three different non-White ethnic groups, perceptions of school and peer racial climate are increasingly positive as schools become *less* diverse. However, we cannot draw firm conclusions for the entire student body from these studies as they did not include White students. One of the few studies actually investigating the effects of school diversity on emotional outcomes among students from both minority and majority students has been conducted by Juvonen et al. (2018). These authors reported that as school ethnic diversity increased, all students - regardless of which ethnic group they belonged to - reported feeling less victimized, less lonely, and safer at school. With higher diversity, students further scored their teachers higher on an index of fair and equal treatment of students. Hence, the aforementioned studies revealed that school diversity seems to generally yield positive effects on well-being.

In the present Study, we want to further probe the main effect of school diversity, as well as its cross-level moderation effect in the relationship between the social support granted by parents, teachers and friends on the one hand and well-being on the other.

*Hypothesis 3*: There will be a positive effect of school diversity on school well-being.

- Research question 4: How does school diversity affect the relationship between perceived support (teacher, parental and peer) and both types of well-being?

## 2. The present study

**Figure 1:**
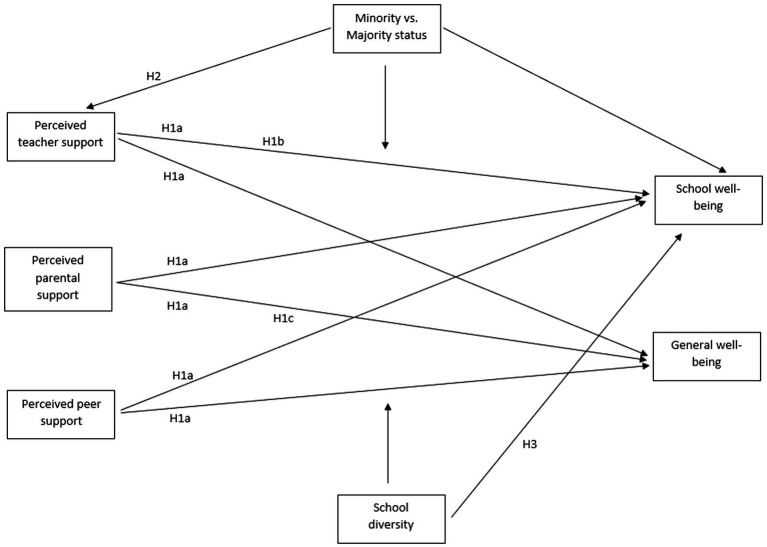
Paths between perceived social support and well-being, moderated by minority status and school diversity.

In the present study, we aimed to examine the relationship between perceived social support and general and school well-being, as well as the moderating role of minority status and diversity in these associations. Figure 1 shows our hypothesized model. We start by looking into the relationship between perceived social support and school well-being and general well-being (i.e., Hypotheses 1a-c). Next, we investigate the main effects of minority status on all support variables and school well-being (i.e., Hypothesis 2 and Research question 1 and 2), as well as potential moderating effects of minority status on the aforementioned relationship (i.e., Research questions 3). Lastly, we examine the main effect of school diversity on school well-being (i.e., Hypothesis 3), and we explore how school diversity may moderate the relationship between perceived social support and both types of well-being (i.e., Research question 4).

The materials, data files, and data scripts of this study can be accessed through our Open Science webpage^1^. Our study was approved by the Ethical Committee of our faculty.

## 3. Materials and methods

### 3.1. Participants

The original sample consisted of 13,871 children from 163 primary schools located in the Belgian province of Antwerp. One student was removed because he or she did not fill in his class and school information correctly. The 163 schools comprised a total of 754 classes (4th, 5th and 6th year, ages 9–12). Mean age was 10.03 years (SD = 0.944). Virtually identical numbers of boys (50.4%) and girls (49.6%) participated, but due to an administration error gender was not successfully registered for part of the sample (39.1% of the sample).

Given that Hypothesis 2 focused on a formal comparison of Belgian majority youth and minority youth, we decided to limit our scope for the minority youth to pupils which could easily and unambiguously be classified as belonging to either the Middle Eastern minority group or the Eastern European minority group. Both groups could easily be identified from the data using the question “which language do you speak at home?” We refer to Valcke et al. (2022) who used an identical methodology and classification. Middle Eastern minority pupils were classified as such when participants indicated to speak one of the following nine languages at home: Arabic, Turkish, Urdu, Pakistani, Afghan, Kurdish and Persian. These languages are spoken in predominantly Middle Eastern countries (Infoplease, n.d.). A total of *n* = 1,136 pupils was assigned to the Middle Eastern minority group. Furthermore, participants who indicated to speak one of the following languages at home, were classified as Eastern European minority group members: Armenian, Bulgarian, Georgian, Hungarian, Yugoslavian, Macedonian, Montenegrin, Ukrainian, Polish, Rumanian, Russian, Serbian, Slovakian, Czech and Croatian. A total of *n* = 434 pupils was assigned to the Eastern European group. The total number of pupils of Belgian descent was *n* = 10,645. A further *n* = 1,656 were not retained in our final analyses, because they could not unambiguously be classified as belonging to a specific cultural group based on the language question. For a comprehensive overview of the used classification system, we refer to Valcke et al. (2022). As such, our final sample comprised *N* = 12,215 (87.1% Belgian majority, 9.3% Middle Eastern minority, 2.6% Eastern European).

### 3.2. Procedure

Data collection was done by the Welfare and Health Department of the Province of Antwerp. Based on the list on the website of the Ministry of Education, all primary schools of the Province of Antwerp (*N* = 583), were contacted and invited to register for participation. The schools that accepted the invitation (*N* = 163) were sent a link to the online questionnaire together with the test instructions. A sample letter for the written consent of the parents was included. The questionnaire consisting of 72 questions was administered between mid-October and early December 2016. To test for the comprehensibility of the items included in the questionnaire, a pilot session was done in a primary school in Beveren in the province of East Flanders.

### 3.3. Measures

Table 1 provides an exhaustive overview of reliability and validity statistics (Cronbach’s Alpha, McDonald’s Omega, Composite Reliability and Average Extracted Variance) for each measure of the focal variables.

**Table 1:** Overview of reliability and validity statistics (Cronbach’s alpha, McDonald’s omega, composite reliability and average extracted variance) for measures of the focal variables.

	*α*	*ω*	CR	AVE
1 SWB	0.71	0.76	0.51	0.56
2 GWB	0.75	0.85	0.59	0.67
3 PPaS	0.79	0.82	0.55	0.51
4 PPeS	0.83	0.86	0.62	0.44
5 PTS	0.81	0.84	0.59	0.46

α, Cronbach’s alpha; ω, McDonald’s omega; CR, composite reliability; AVE, average extracted variance; 1 SWB, school well-being; 2 GWB, general well-being; 3 PPaS, perceived parental support; 4 PPeS, perceived peer support; 5 PTS, perceived teacher support. Combined values of CR > 0.60 and AVE > 0.40 indicate acceptable reliability and validity (Fornell and Larcker, 1981).

#### 3.3.1. Social support

Support from different sources was measured by four questions. Answers to the first three questions (“If I have a problem, the following people would give me good advice,” “If I am sad, the following people would comfort me,” and “If I have a problem, the following people would do something to help me”) were given on a 5-point Likert scale (0 = certainly not, 1 = probably not, 2 = maybe, 3 = probably, 4 = certainly) for three sources of support, namely “mother/father,” “friend,” “teacher.” The fourth item (“If I have problems, I can go to”) was answered on a 5-point Likert scale (0 = never, 1 = almost never, 2 = sometimes, 3 = usually, 4 = always) for six sources of support, among these “mother/father,” “teacher” and “friend.” We calculated a mean support score for parents (*M* = 3.59, *SD* = 0.60), friends (*M* = 3.01, *SD* = 0.80), and teachers (*M* = 3.00; *SD* = 0.80).

#### 3.3.2. School well-being

We administered four items to measure school well-being. The first item probed into the extent to which the child generally liked going to school, scored on a 5-point Likert scale (0 = do not like to go at all, 1 = do not like to go, 2 = like to go a little, 3 = like to go, 4 = like to go very much). The second item was about how they usually feel about recess, answered on a 5-point Likert scale (0 = not fun at all, 1 = not fun, 2 = a little fun, 3 = fun, 4 = a lot of fun). The third and fourth items, respectively, probed into (1) how they usually felt about classes at school and (2) about homework on a 5-point Likert scale (0 = not fun at all, 1 = not fun, 2 = a little fun, 3 = fun, 4 = a lot of fun). We calculated school well-being by averaging these four items (*M* = 2.69; *SD* = 0.69).

#### 3.3.3. General well-being

This variable was measured with a composite index of two scales which have frequently been used in literature to study well-being, experienced happiness and positive/negative affect. Two questions measured experienced happiness (Lyubomirsky and Lepper, 1999; Bruggeman et al., 2019), asking how happy participants usually felt and how happy they had felt the day before they took the survey. Answers were given on an 11-point Likert scale (ranging from 0 = very unhappy to 10 = very happy). The average score in the sample on the first question (“usually”) was 7.96 (*SD* = 1.77), the average score on the second question (“yesterday”) was 8.17 (*SD* = 2.22). The two items were significantly related, *r* = 0.53, *p* < 0.001. Furthermore, eight items from the Positive and Negative Affect Schedule for Children (PANAS-C, Laurent et al., 1999; translated by De Bolle et al., 2010) were also selected. Pupils were asked to indicate how often they had experienced four positive emotions during the past week, namely “happy,” “full of energy,” “proud,” and “happy,” and four negative emotions, namely “lonely,” “sad,” “anxious,” and “unhappy,” to measure positive and negative affect, respectively. A 5-point Likert scale was used for each emotion (0 = never, 1 = almost never, 2 = sometimes, 3 = usually, 4 = always, positive affect: *M* = 3.03, *SD* = 0.58, *α* = 0.658; negative affect: *M* = 0.81, *SD* = 0.67). The negative affect items were recoded to create a full ‘positive affect’ score.

We calculated general well-being by averaging the mean scores on the two individual measures (*α* = 5.61, *M* = 1.03).

#### 3.3.4. School diversity

We created an ethnic diversity score D per school based on the diversity of languages students indicated speaking at home. This was done by calculating “The Simpson’s diversity index” (Budescu and Budescu, 2012; Simpson, 1949). The following formula was used:


$$D=1-\frac{\Sigma n\left(n-1\right)}{N\left(N-1\right)}$$

With *n* = the total number of students speaking a particular language in a given school, and *N* = the total number of students in that school. This index measures the probability that two individuals randomly selected from a sample will belong to different categories – which, in the current study, entailed the probability that two students randomly selected from the same school would speak a different language at home. The values of D can range from 0 to 1, with 0 indicating ‘no diversity’ and 1 representing ‘absolute diversity’. To calculate D, we used the full sample – i.e., including the *n* = 1,656 participants which were excluded from our final analyses. We did so because, although these participants could not unambiguously be classified as belonging to either one of our three specific minority groups, they nonetheless obviously contribute to diversity. The mean school diversity in our sample was 0.35 (*SD* = 0.18). Furthermore, school diversity ranged from 0 (uniform distribution of spoken languages) to 0.83 (very high chance of randomly selecting two pupils speaking different languages at home).

## 4. Data analyses and results

### 4.1. Preliminary analyses

Pearson correlations among the study’s variables are reported in Table 2.

**Table 2:** Pearson correlations among our study’s focal variables.

		1	2	3	4	5
1 SWB		-				
2 GWB		0.41**	-			
3 PPaS		0.21**	0.30**	-		
4 PPeS		0.26**	0.31**	0.29**	-	
5 PTS		0.38**	0.26**	0.38**	0.38**	-

*N* = 13,870. 1 SWB, school well-being; 2 GWB, general well-being; 3 PPaS, perceived parental support; 4 PPeS, perceived peer support; 5 PTS, perceived teacher support. **p < 0.01.

Initial analyses revealed significant gender differences for all three types of perceived social support (teacher: *M*b_oys_ = 2.97, *SD*_boys_ = 0.83, M_girls_ = 3.07, *SD*_girls_ = 0.71; *F*(1, 7,439) = 31.61, *p* < 0.001; parental: *M*b_oys_ = 3.60, *SD*_boys_ = 0.56, M_girls_ = 3.62, *SD*_girls_ = 0.57; *F*(1, 7,422) = 4.13, *p* = 0.042; peers: *M*b_oys_ = 2.86, *SD*_boys_ = 0.82, M_girls_ = 3.20, *SD*_girls_ = 0.71; *F*(1, 7,422) = 356.15, *p* < 0.001), and school well-being (*M*b_oys_ = 2.58, *SD*_boys_ = 0.72, M_girls_ = 2.79, *SD*_girls_ = 0.64; *F*(1, 7,487) = 178.52, *p* < 0.001) and general well-being (*M*b_oys_ = 5.64, *SD*_boys_ = 1.01, M_girls_ = 5.56, *SD*_girls_ = 1.07; *F*(1, 7,487) = 11.44, *p* < 0.001). Furthermore, we also found significant relationships between age and all the focal variables (teacher support: *r* = −0.04, *p* < 0.001; parental support: *r* = 0.04, *p* < 0.001; peer support: *r* = 0.08, *p* < 0.001; school well-being: *r* = −0.10, *p* < 0.001), except general well-being (*r* = 0.00, *p* = 0.990). As such, we decided to retain these demographic covariates in our main analysis.

### 4.2. Main analyses

To satisfy our main research aims, we fitted a single (generalized) linear mixed model (GLMM) with the Lavaan package (Rosseel, 2012) in R (R Core Team, 2022), with all three forms of perceived support as independent variables, and general well-being and school well-being as outcomes. Minority status (Belgian majority youth/Middle Eastern minority youth/Eastern European minority youth) and school diversity (operationalized by the Simpson index) were included as moderating variables, gender and age were included as covariates. In an additional analysis we also included interaction terms between gender and age and the focal predictors (perceived support, minority status and school diversity). Given that these interactions were all found to be insignificant, we decided to report the output of the more parsimonious model below (i.e., with only main effects for gender and age). Missing data were handled within the analysis model, by means of Full Information Maximum Likelihood Estimation. The model output (parameter estimates, standard errors (*SE*s), significance tests and 95% confidence intervals [CIs]) is displayed in Table 3. In what follows, we present a brief overview of the most important findings for both forms of well-being.

**Table 3:** Results of linear mixed model with perceived social support variables, minority status and school diversity (Simpson index) as predictors, and school well-being and general well-being as outcomes [parameter estimates (unstandardized betas), SEs, 95% confidence intervals (CIs between parentheses) and significance tests are reported].

Outcome variable								
I. School well-being					II. General well-being			
Fixed Effects	*B* (*SE*)	95% CI	*p*	*R* ^2^	*B* (*SE*)	95% CI	*p*	*R^2^*
PTS	**0.28 (0.027)**	**[0.232;0.337]**	**<0.001**	**0.019**	**0.13 (0.050)**	**[0.031;0.228]**	**0.010**	**0.002**
PPaS	0.08 (0.039)	[−0.001;0.151]	0.053	0.001	**0.35 (0.068)**	**[0.220;0.488]**	**<0.001**	**0.006**
PPeS	**0.10 (0.027)**	**[0.046;0.151]**	**<0.001**	**0.002**	**0.27 (0.047)**	**[0.178;0.363]**	**<0.001**	**0.007**
**Minority status**								
Middle Eastern	0.30 (0.187)	[−0.065;0.670]	0.107	0.001	0.64 (0.341)	[−0.025;1.313]	0.059	0.001
Eastern European	−0.09 (0.293)	[−0.665;0.483]	0.483	0.000	**0.99 (0.456)**	**[0.094;1.881]**	**0.030**	**0.001**
School diversity	0.38 (0.416)	[−0.440;1.190]	0.367	0.000	−0.11 (0.605)	[−1.292;1.081]	0.862	0.000
PTS*Middle E.	−0.02 (0.047)	[−0.114;0.070]	0.637	0.000	−0.05 (0.059)	[−0.169;0.062]	0.361	0.000
PTS*Eastern E.	**−0.18 (0.063)**	**[−0.300;-0.052]**	**0.005**	**0.001**	**−0.22 (0.089)**	**[−0.390;-0.041]**	**0.016**	**0.001**
PPaS*Middle E.	**0.13 (0.057)**	**[0.016;0.238]**	**0.025**	**0.001**	−0.07 (0.100)	[−0.264;0.128]	0.497	0.000
PPaS*Eastern E	**0.19 (0.077)**	**[0.038; 0.341]**	**0.014**	**0.001**	−0.04 (0.133)	[−0.296;0.223]	0.784	0.000
PPeS*Middle E.	**−0.16 (0.039)**	**[−0.237;-0.082]**	**<0.001**	**0.000**	−0.06 (0.057)	[−0.174;0.050]	0.275	0.000
PPeS*Eastern E.	0.04 (0.046)	[−0.045;0.133]	0.334	0.000	−0.02 (0.104)	[−0.221;0.188]	0.877	0.000
PTS*diversity	0.00 (0.080)	[−0.152;0.160]	0.959	0.000	−0.01 (0.141)	[−0.281;0.271]	0.973	0.000
PPaS*diversity	−0.10 (0.118)	[−0.333;0.130]	0.389	0.000	−0.02 (0.187)	[−0.388;0.344]	0.907	0.000
PPeS*diversity	0.02 (0.070)	[−0.120;0.155]	0.804	0.000	0.04 (0.121)	[−0.193;0.281]	0.717	0.000
**Gender**	**0.15 (0.017)**	**[0.115;0.181]**	**<0.001**	**0.013**	**−0.19 (0.028)**	**[−0.244;-0.135]**	**<0.001**	**0.009**
**Age**	**−0.08 (0.011)**	**[−0.102;-0.059]**	**<0.001**	**0.014**	**−0.03 (0.015)**	**[−0.060;-0.001]**	**0.044**	**0.001**

*N* = 12,215. PPaS, perceived parental support; 4 PpeS, perceived peer support; 5 PTS, perceived teacher support. Minority status was dummy-coded (Belgian majority group as reference category). Controlled for gender (dummy-coded; 1 = girls, 0 = boys) and age. *R*^2^ = proportion of unique variance explained in outcome by individual predictor. Model *R*^2^ = 0.197. Significant effects are printed in bold.

#### 4.2.1. The relationship between perceived support and school and general well-being

The results revealed significant main effects of sources of social support on school well-being (teacher support: *b* = 0.28, *β* = 0.33, *p* < 0.001; peer support: *b* = 0.10, *β* = 0.11, *p* < 0.001), except for parental support (*b* = 0.08, *β* = 0.06, *p* = 0.053). These results thus partially supported the first part of Hypothesis 1a, which stated that all three sources of perceived support would be positively related to school wellbeing.

To formally investigate Hypothesis 1b, which stated that the relationship between perceived social support and school well-being is the strongest for teacher support, we performed a series of general linear hypothesis tests using the *ghlt* function from the *multcomp* package (Hothorn et al., 2008) in R. In each of these tests, the relative magnitude of the coefficient of the perceived teacher support-school well-being relationship was pitted against the magnitude of one of the other two relevant regression weights [i.e., *β* (peer support-school well-being) and *β* (parental support-school well-being)], by investigating the null hypothesis that their linear sum is equal to 0. Rejection of this null hypothesis was then indicative of a larger regression coefficient for teacher support – and hence a stronger association between teacher support and school well-being (compared to the other social support variable).

The results of this analysis revealed that the regression coefficient for the relationship between perceived teacher support and school well-being was significantly larger than that of the peer support-school well-being (∆ = −0.18, *p* < 0.001) and parental support-school wellbeing relationships (∆ = −0.20, *p* < 0.001). The peer support-school well-being and parental support-school well-being relationships did not differ in magnitude (∆ = −0.02, *p* = 0.645) These results thus supported Hypothesis 1b,.

Significant main effects were also found for all three support variables on general well-being (all *b*s > 0.12, all, *β*s > 0.09, all *p*s < 0.011). These results thus supported the second part of Hypothesis 1a, which stated that all three sources of perceived support would be positively related to general wellbeing. Further comparison of regression coefficients – using the same procedure as above – unraveled that the regression coefficient for the relationship between perceived parental support and general well-being was significantly larger than that of the teacher support-general well-being relationship (∆ = 0.20, *p* = 0.005). Likewise, the regression coefficient for the relationship between perceived peer support and general well-being was significantly larger than that of the teacher support-general well-being relationship (∆ = 0.13, *p* = 0.038). The parental support-general well-being and peer support-general wellbeing relationships did not differ in magnitude (∆ = 0.08, *p* = 0.281). These results did thus not fully corroborate Hypothesis 1c.

#### 4.2.2. The relationship between minority status and perceived social support

To jointly investigate Hypothesis 2 (which stated that children of cultural minority groups will report lower perceived teacher support than their majority counterparts) and Research question 1 (which inquired about potential effects of cultural minority status on perceived peer and parental support), we fitted a linear mixed model with source of support (teacher, parental and peer) and minority status (Belgian majority, Middle Eastern minority and Eastern European minority) as predictors, and perceived support as the dependent variable. Random subject-specific and school-specific effects were added to account for the nested structure of our data.

The results of this analysis revealed significant main effects of source of support (*F*(2, 24,282) = 858.17, *p* < 0.001) and minority status (*F*(2, 12,204) = 73.71, *p* < 0.001). These main effects, however, were further qualified by a significant source of support x minority status interaction (*F*(4, 24,724) = 14.10, *p* < 0.001). To further probe this interaction, we ran a series of contrasts, using the *emmeans* package (Lenth, 2022) in R. Specifically, we calculated the mean associated with each source of support-cultural group combination, and we compared these means in a pairwise fashion (using Tukey’s method to adjust for multiple comparisons). The results of these analyses are depicted in Table 4.

**Table 4:** Overview of results of planned contrasts comparing three types of perceived social support (teacher, parents and peers) as a s function of minority status (Belgian majority group vs. middle eastern minority vs. eastern European minority).

	∆(BE - ME)	∆(BE - EE)	∆(ME - EE)
1. Teacher support	0.03	0.08	0.04
2. Parental support	0.17***	0.20***	0.03
3. Peer support	0.20***	0.21***	0.01

*N* = 12,251. BE, Belgian majority group; ME, middle eastern minority group; EE, eastern European minority group. Significant differences between cultural groups are printed in bold. ***p < 0.001.

A closer look at this table reveals no significant differences in perceived *teacher support* between the three cultural groups (Belgian majority, Middle Eastern minority and Eastern European minority; all ∆s < 0.09, all *p*s > 0.324). Conversely, significant differences were found between the Belgian majority group and both cultural minority groups for perceived *parental* (both ∆s > 0.16, both *p*s < 0.001) and *peer support* (both ∆s > 0.19, both *p*s < 0.001). The Middle Eastern minority and Eastern European minority group did not differ significantly from one another for both types of perceived support (both ∆s < 0.05, both *p*s > 0.993). These results thus do not provide any evidence for Hypothesis 2, which stated that children of minority groups will report lower perceived teacher support than their majority counterparts. Moreover, they also provide an answer to Research question 1, which focused on possible effects of minority status on perceived peer and parental support. That is, our results indicate that minority group pupils are less likely to report receiving support from parents and peers than majority group students.

#### 4.2.3. Main and moderating effects of minority status on school well-being

A closer look at Table 3 reveals no significant main effect of minority status on school well-being (both *p*s > 0.106) – a finding which runs counter to Research question 2 (i.e., pupils of cultural minority groups will report higher school well-being, compared to majority group pupils). These results, however, were further qualified by a few significant minority status x social support interactions. (1) Middle Eastern (*b* = 0.12, *β* = 0.19, *p* = 0.025) and Eastern European minority pupils (*b* = 0.19, *β* = 0.18, *p* = 0.014) both showed a stronger relationship between perceived parental support and school well-being, compared to the Belgian majority group. (2) The Middle Eastern minority group also reported a weaker relationship between perceived peer support and school-wellbeing (compared to Belgian majority pupils; *b* = −0.16, *β* = −0.20, *p* < 0.001). (3) A weaker relationship between perceived teacher support and school-wellbeing was found for the Eastern European minority group, compared to the Belgian majority group (*b* = −0.18, *β* = −0.14, *p* = 0.005).

Taken together, these results thus address the issue raised in Research questions 3 (i.e., does minority status affect the relationship between perceived support and school well-being?). Specifically, it was shown that minority students belonging to both cultural minority groups benefited more from parental support in terms of school well-being. Conversely, the benign effects of teacher and peer support on school well-being were somewhat curbed among Eastern European and Middle Eastern pupils, respectively.

#### 4.2.4. Main and moderating effects of school diversity on well-being

No significant main effects of school diversity on the well-being variables were found (both *p*s > 0.366), nor any cross-level interactions between diversity and all sources of perceived social support (all *p*s > 0.388). These results thus failed to corroborate Hypothesis 3 – which stated that there would be a positive effect of school diversity on school well-being. Moreover, given that we failed to obtain any significant moderation effects, these findings also inform Research question 4, which inquired if school diversity affects the relationship between perceived support and both types of well-being.

## 5. Discussion

The importance of perceived social support has been recognized for several school-related outcomes like academic achievement (Ahmed et al., 2010), school dropout (Lagana, 2004), academic adjustment (Malecki and Demaray, 2007) and student burnout (Meylan et al., 2015). The present study’s primary aim was to add to this literature by investigating the relationship between perceived social support and well-being, taking into account ethnic-cultural background and school diversity. Moreover, the present study also aimed to provide a more fine-grained understanding of this association, by disentangling the unique contributions of various types of support (teacher, parental, and peer) on two different types of well-being (school and general) – thereby also isolating the unique effects on school well-being as such.

Another important novelty of the present research is that we have differentiated between two groups of minority pupils. This distinction between two minority groups allows offer a more nuanced view and underlines the necessity to acknowledge the possible differences between minority groups. Especially with respect to the European context, minorities differ in terms of regional descent, which may be at the basis of noteworthy differences. Due to our large sample size, thus, we were able to compare Middle Eastern minorities and East-European immigrants.

### 5.1. Perceived teacher support among minorities and majorities

The results of our study both replicate and nuance previous research findings. In line with Hypothesis 1a, our results show that all three sources of perceived support are positively related to school wellbeing and general well-being. In addition, and in line with Hypothesis 1b, it was found that, in general, the relationship between perceived social support and school well-being is the strongest for teacher support, compared to parental support and peer support. Our results match with those of the previous studies, revealing that, in general, teacher support is the most important source for school well-being (Rosenfeld et al., 2000; Ramos-Diaz et al., 2016). Lastly, in contrast to what was expected in Hypothesis 1c, parental support did not show the strongest relationship with general well-being (compared to teacher support and peer support). In contrast to the findings of Chu et al. (2010), peer support seemed to be an equally important source of support for general well-being as parental support. The relationship between teacher support and general well-being showed to be weaker and thus less crucial.

The present research did not obtain supportive evidence for Hypothesis 2 (i.e., children of minority groups will report lower perceived teacher support) Indeed, we could not find any significant differences in perceived teacher support between the three cultural groups in this study – at least, not when type of support was entered into the equation. This result thus does not corroborate studies conducted in the North American context indicating that children of minority groups have less positive relations with their teachers (Hamre and Pianta, 2001; Hughes and Kwok, 2007). A few tentative explanations for this discrepancy can be envisaged. For example, it can be argued that the European and American educational systems differ. Furthermore, the minority groups and their migration background in our Belgian sample are substantially different from those studied in previous investigations conducted in North-America. Indeed, the studies conducted in the United States have mainly focused on African American children as minority group members, whereas we investigated Middle Eastern and Eastern European minority pupils. Unlike the American context, the migration waves of these minorities to Europe have begun only recently, 50 years ago. Moreover, this type of migration has continued up to today. The parents of the children under study in the present investigation can therefore show differing levels of integration in the host culture, and may have different levels of acquaintance with the educational system. Such unfamiliarity with the Belgian school system may have culminated into lower levels of expected support – compared to the US situation – and may consequently have elevated the perception of support when it was *de facto* provided by the teachers. However, a most marked difference between the present study and previous investigations is the inclusion of pupils pertaining to a younger age group, which distinguishes it from those studies conducted in the United States which investigated secondary school pupils. Hence, one possibility we cannot exclude is that the negative perceptions of teacher relationship and support might grow over the years, and thus becomes negative only in secondary school. Follow-up studies in the United States on primary school samples is thus needed to know if the previously found ethnic-cultural differences in the perception of teacher support are already cultivated at a younger age.

Moreover, the results did provide an answer for Research question 1. The results showed significant differences between the Belgian majority group and both cultural minority groups for perceived parental support and peer support, showing that minority members report lower feelings of both sources of support compared to the majority group. Both minority groups did not differ from each other. Through this lens, in relative terms, the minority pupils in the present sample thus have rated teachers support at a high level, as compared to parents and peer support. In other words, even though minority pupils were inclined to feel less support of parents and peers, they did not experience less support from teachers. From this it can in fact be concluded that teachers elicited a fairly high level of *perceptions* of social support among their pupils – at least, relative to the other focal socialization figures (parents and peers).

### 5.2. The relationship between social support and well-being: Differences between ethnic-cultural groups?

With respect to Research question 2, no significant main effect was obtained of minority status on school well-being. However, with regard to Research question 3, our results revealed a weaker relationship between perceived teacher support and school well-being for Eastern European pupils, compared to the majority group. This finding runs counter to previous studies showing no significant moderating effect of minority status on the teacher support-school well-being relationship (e.g., Bird and Markle, 2012; Griffith, 2002). Furthermore, this results does not mirror our observations among Middle Eastern minority group pupils, who benefited from teacher support (in terms of school well-being) to a similar extent as majority group pupils. One potential explanation for this finding may reside in the fact that the Eastern European minority group constitutes a relatively “new” migration group, compared to the Middle Eastern group (Myria, 2016). Although migration from countries like Poland to Belgium has remained quite stable during the 20^th^ century, migration waves from various other Eastern European nations have only accelerated over the last two decades. In this respect, they clearly differ from the Middle Eastern group, who are in fact often second-or even third-generation migrants. As such, there may be various other barriers for Eastern European minority pupils – e.g., unfamiliarity with the Belgian educational system, language barriers, etc. – that prevent teacher support from enhancing school well-being. Further research incorporating these variables is needed to answer these questions.

Secondly, the results showed that for both minority groups, perceived parental support was more strongly related to school well-being, compared to the Belgian majority group. A similar relation has been shown with academic achievement as an outcome variable (Ceballo et al., 2014). Specifically, Ceballo et al. (2014) revealed that the positive relation between parental involvement and academic achievement was stronger for immigrant youth. Our results add to these scholars’ findings the notion by showing that minority pupils are not only more responsive to parental support when it comes to school achievement, but also in terms of school well-being.

In addition, for Middle Eastern minority group pupils, a weaker relationship between perceived peer support and school well-being was also found, compared to the majority group. An explanation this weaker relationship may be that students’ peers are part of a different group. Minority students are clearly outnumbered by Belgian majority students in our sample, and we therefore can assume that most pupils have a number of cross-ethnicity friendships in their school social network. Especially for the Middle Eastern pupils, this often entails interactions with pupils that have very different cultural customs and traditions. If these relationships would be less harmonious than same ethnicity relationships, then support perceptions may drop. However, as we did not administer the ethnicity of the peer social network in the present study, these hypotheses await further empirical test.

### 5.3. School diversity

Hypothesis 3 stated that school diversity would have beneficial effects on school well-being. Hence, besides the effect of individual-level minority status on individual perceived support and reported well-being, we also looked into the cross-level effect of school-level diversity. School diversity was calculated based on students’ home language using the Simpson index. To the best of our knowledge, only two previous studies investigating school well-being have used the Simpson index as a measure of school diversity, and have included both cultural minority and cultural majority groups in the same sample. The first of these studies has shown that higher school diversity yields positive effects, because it is associated with feeling less lonely, less victimized, and safer, regardless of students’ ethnicity. Thus, the more equally each ethnic group is represented in a school, the better students feel (Juvonen et al., 2018). The second study points towards similar benign effects of school diversity – at least for White majority students. Specifically, school well-being (operationalized as suspension rates) was significantly and negatively associated with school diversity among White (but not Black) students (Bottiani et al., 2016). Our results, however, failed to corroborate these prior studies and Hypothesis 3: No significant main effects of school diversity on the well-being variables were found. In addition, in answer of Research question 4, no significant moderation effects were found, indicating that school diversity did not affect the relationship between perceived support (teacher, parental and peer) and both types of well-being. One potential explanation for these unexpected results may be that school diversity was less “visible” in our study, compared to the work described above. That is, school diversity has been characterized in these studies in terms of the presence of members of clearly distinct and salient racial groups – for example in terms of skin tone (e.g., Black, Latino, Asian minority pupils). Conversely, in the present research, school diversity was operationalized in terms of the number of languages spoken at home. It stands to reason that language diversity is a less salient school attribute than racial/ethnic diversity, and the former type of diversity may simply not be sufficient to yield the previously observed benign effects. Another possible explanation for our diverging findings is that, although school diversity was found to be medium-to-high in general (i.e.,. D = 35), this substantial average diversity did not translate into equality in numbers of the various (language) groups. Indeed, in our sample, certain groups were clearly overrepresented (e.g., Dutch-speaking, Turkish-speaking), whereas others were noticeably underrepresented (e.g., Nepali-speaking, Finnish-speaking). As Juvonen et al. (2018) observed, equal group representation is particularly key for school well-being, and these intergroup differences in terms of group size may thus have further prevented school diversity to yield its purported positive effects. Further research implementing alternative diversity indicators could help clarify such questions.

### 5.4. Other demographic variables

We have now only discussed the effects of students’ minority status on perceived social support and well-being. However, we also included gender and age as control variables in our study, as well as the interaction effects of gender and age with minority status. Girls perceive more support and report higher levels of school well-being, but lower levels of general well-being. Our results also show that girls especially perceive greater teacher and peer, and to a lesser extent – but still significantly – more parental support. This replicates results of previous studies showing that girls report higher levels of teacher (Ellonen et al., 2008) and peer (Ellonen et al., 2008; Rueger et al., 2010) support. With regard to parental support, our results did not corroborate a previous study of Helsen et al. (2000) who showed that there are no differences in perceived parental support between boys and girls. The difference in perceived teacher support may be partially explained by the interaction style from teachers towards students, which may generally include more disciplinary actions towards boys than towards girls, which in turn might cause boys to perceive teachers as less supportive (Finn and Rock, 1997). Because of such an interaction style, boys may also perceive less parental support. However, this would not explain the finding of lower perceived peer support in boys. Another, possibly additional, explanation of our results can be found in the fact that boys and girls share different beliefs about communality (i.e., the needs and desires of the interaction partner determine social behavior) versus exchange (i.e., social behavior is determined by the comparability of benefits for each interaction partner) in relationships, and that these different beliefs might influence perceptions of support (Murstein and Azar, 1986; Jones and Costin, 1995). It has been shown that girls cherish communality beliefs more than boys, which relates positively to perceptions of support. In contrast, boys score higher on exchange beliefs than girls, which relates negatively to support perceptions (Colarossi and Eccles, 2003).

We also obtained some interesting age effects. Specifically, both types of well-being declined with increasing age, and we noted a shift in the importance of support sources as children grow older. Previous studies suggest that the importance of the different support sources varies with age (e.g., Tian et al., 2015; Wang and Eccles, 2012) indicating a shift in social priorities from adults to peers happening in adolescence (Wang and Eccles, 2012). In the present study, the effects of age were rather modest, but still we found a similar pattern in a sample of children with an average age as young as 10.3 years. Specifically, whereas perceived peer and parental support increased with a small but significant effect, a decrease in perceived teacher support with age was also observed. One possible explanation for this decrease might be the growing need for autonomy as children grow older, combined with the authority roles of teachers (e.g., Claes et al., 2001; Cattley, 2004).

Finally, The present results revealed that the effects of minority status on school well-being were not significantly moderated by gender and age, implying that the effects of minority status applied to an equal extent for boys and girls, and for younger and older pupils A last interesting result with regard to the demographic variables, is that teacher support had its most beneficial effects on school well-being for boys. As we argued above, the interaction style towards boys, with more disciplinary actions towards boys, lead to relatively low levels of perceived teacher support. Because of this low level of perceived teacher supports, incremental gains in this type of support may have greater effects.

In terms of practical implications, these latter results seem particularly relevant for both educational practitioners and policy-makers in Belgium. On the one hand, teachers may have to be attentional to support male pupils (in general, not only those with an ethnic minority background) in a sufficient manner, since this group may be most beneficially influenced by these acts of support. Additionally, policy-makers should pay extra attention to the decrease in well-being with increasing age, and how this decline may be buffered in a school context.

### 5.5. Limitations

The present research also suffers from some potential limitations. For example, it must be noted that we only sampled primary school pupils pertaining to two ethnic-cultural minority groups. Given that, in the Belgian context, diversity is characterized by the presence of a plethora of ethnic-cultural groups, it seems conceivable that the lack of any main effects of minority status may be an artifact of the absence of other ethnic-cultural groups in our research design. Future studies could circumvent this limitation by focusing on the perspective of a wider array of ethnic-cultural minority groups.

Another possible limitation is that we only had information on the languages the children in our sample spoke, but not on where they and their parents were born. A further challenge for the categorization by language is the fact that pupils who indicated to speak only Dutch at home were automatically categorized in the ‘majority group’. It is however, possible that some of these pupils actually belong to a family of third or fourth generation immigrants in Belgium^2^. We here want to refer to the formal definition that is often used in research using Belgian samples to classify ethnic minorities. Specifically, the definition used to categorize people as ‘allochthonous’ pertains to persons residing in Belgium, regardless of whether they possess Belgian nationality, at least one of whose parents or grandparents was born outside Belgium - commonly outside West Europe - and who have a disadvantaged position in society because of their ethnic origin (Brans et al., 2004). Following this definition, one is not considered to be “allochthonous” anymore when he/she belongs to the fourth generation. Actually, this definition implies that people from the fourth generation do not differ much from native Belgians and for this reason they are not categorized as ethnic minorities anymore. We have cross-validated the present language variable in another large sample where we also asked the participants to indicate whether they have a (grand)parent who was born in a non-West European country. From the 3,745 participants who indicated to speak Dutch exclusively at home, only 70 (or 1.87%) indicated to have a (grand)parent born in a Middle Eastern country. Hence, we believe that the number of wrongly classified Middle Eastern pupils is limited and unlikely to have a large influence on the results. However, future studies should supplement the language use variable with direct questions about the migration history of their parents.

Related to the previous point, it should be stated that we did not have any information about how our primary school pupils identified in terms of minority group membership (and thus on how the participants socially identify with the cultural group in which they are categorized). In this vein, it should be stressed that reliance on linguistic characteristics to determine whether a given participant is a minority group member or not can be tedious, and this aspect of our research design could have obscured factual differences between the studied ethnic-cultural groups. We therefore strongly encourage future researchers to investigate the effects of minority status on school well-being by operationalizing minority group membership in terms of how the youth population under study self-identifies, in order to scrutinize the robustness of the present results.

Finally, although our findings with respect to the moderating role of minority status are interesting in their own right, they should nonetheless be interpreted with caution. That is, a closer look at Table 3 also reveals that, following Cohen (1988); pp. 413–414) classification of magnitudes of *R^2^* effect sizes (i.e., “small” = 0.02, “medium” = 0.13, “large” = 0.26), the effect sizes associated with each of these interactions were all rather small (i.e., *R*^2^s < 0.01). In this regard, it should be noted that our sample size was quite substantial, and, as Hayat (2010) notes, “An increasingly large sample size yields a decreasingly smaller *p* value, [sometimes] regardless of scientific importance.” In other words, given that large sample sizes can inflate *p*-values – and hence, lead to overestimation of the practical significance of an effect – we must thus refrain from drawing strong conclusions based on these observed, yet rather small effects. Further research is needed to substantiate the robustness of the moderating role of minority status on the relationship between perceived social support and school well-being. In addition, it is noteworthy that the presently obtained effects of minority status are rather modest compared to the magnitude of the effects of gender and age. In primary schools, cultural ethnic background may have more limited effects than in other age groups. One of the interesting avenues for further study, therefore, is to probe into the role of minority status in a sample with a wider age range.

### 5.6. Concluding remarks

The present study investigated the joint effects of perceived social support, minority status, school diversity, age and gender on school and general well-being. As predicted, teacher support emerged as the most relevant source of support predicting school well-being, and peer support was shown to play an equally significant role in general well-being as parental support. Minority status was shown to moderate these associations, although we must nuance these findings because of the relatively small associated effect sizes. School diversity did not yield any relevant effects. Taken together, and given that perceived teacher support was also found to be positively associated (albeit less strongly than peer and parental support) with general well-being, the present results thus highlight the pivotal role that teachers can play in the wellbeing of primary school pupils – both in the education context and beyond – of all ethnic groups.

## Data availability statement

Publicly available datasets were analyzed in this study. This data can be found at: https://osf.io/8vsjn/?view_only=5c046a24cd5c4ce2b869769d56f03acf.

## Ethics statement

The studies involving human participants were reviewed and approved by Ethical commission of the faculty Psychology and Educational Sciences of Ghent University. Written informed consent to participate in this study was provided by the participants' legal guardian/next of kin.

## Author contributions

BV, KD, LD, SD, GH, and AH were involved in the conception and design of the work and the empirical studies. BV provided a first draft of the article. BV, KD, and AH analyzed and interpreted the data and made critical revisions of the article. LD, SD, and GH played a crucial role in the data collection. All authors contributed to the article and approved the submitted version.

## Funding

This work was supported by the Special Research Fund of Ghent University (grant no. BOF16/GOA/007).

## Conflict of interest

The authors declare that the research was conducted in the absence of any commercial or financial relationships that could be construed as a potential conflict of interest.

## Publisher’s note

All claims expressed in this article are solely those of the authors and do not necessarily represent those of their affiliated organizations, or those of the publisher, the editors and the reviewers. Any product that may be evaluated in this article, or claim that may be made by its manufacturer, is not guaranteed or endorsed by the publisher.

## Footnotes

^1^ https://osf.io/8vsjn/?view_only=5c046a24cd5c4ce2b869769d56f03acf

^2^We have reasons to believe that this group is rather small. We base this assumption on another study with a similar sample (primary school pupils from a different province in Belgium). In this sample, the language item was included, but we also asked the participants to indicate whether they have a (grand) parent who was born in a non-West European country. From the 3,745 participants who indicated to speak Dutch exclusively at home (2,569 indicated the country of birth of both their parents and grandparents, the rest of the sample only their parents), only 70 (or 1.87%) indicated to have a (grand) parent born in a Middle Eastern country. Hence, we believe that the number of wrongly classified Middle Eastern pupils is limited and unlikely to have a large influence on the results.
